# Membrane Associated Progesterone Receptors: Promiscuous Proteins with Pleiotropic Functions – Focus on Interactions with Cytochromes P450

**DOI:** 10.3389/fphar.2017.00159

**Published:** 2017-03-27

**Authors:** Chang S. Ryu, Kathrin Klein, Ulrich M. Zanger

**Affiliations:** ^1^Department of Molecular and Cell Biology, Dr. Margarete Fischer-Bosch-Institute of Clinical PharmacologyStuttgart, Germany; ^2^Eberhard-Karls-UniversityTübingen, Germany

**Keywords:** cytochrome P450, membrane-associated progesterone receptor, neudesin, neuferricin, PGRMC1, PGRMC2, protein-protein interaction

## Abstract

Membrane-associated progesterone receptors (MAPR) are a group of four rather small, partially homologous proteins, which share a similar non-covalent heme-binding domain that is related to cytochrome b5, a well-known functional interaction partner of microsomal cytochrome P450 (CYP) monooxygenase systems. Apart from their structural similarities the four proteins progesterone membrane component 1 (PGRMC1, also referred to as IZA, sigma-2 receptor, Dap1), PGRMC2, neudesin (NENF) and neuferricin (CYB5D2) display surprisingly divergent and multifunctional physiological properties related to cholesterol/steroid biosynthesis, drug metabolism and response, iron homeostasis, heme trafficking, energy metabolism, autophagy, apoptosis, cell cycle regulation, cell migration, neural functions, and tumorigenesis and cancer progression. The purpose of this mini-review is to briefly summarize the structural and functional properties of MAPRs with particular focus on their interactions with the CYP system. For PGRMC1, originally identified as a non-canonical progesterone-binding protein that mediates some immediate non-genomic actions of progesterone, available evidence indicates mainly activating interactions with steroidogenic CYPs including CYP11A1, CYP21A2, CYP17, CYP19, CYP51A1, and CYP61A1, while interactions with drug metabolizing CYPs including CYP2C2, CYP2C8, CYP2C9, CYP2E1, and CYP3A4 were either ineffective or slightly inhibitory. For the other MAPRs the evidence is so far less conclusive. We also point out that experimental limitations question some of the previous conclusions. Use of appropriate model systems should help to further clarify the true impact of these proteins on CYP-mediated metabolic pathways.

## Introduction

Inter- and intraindividual variability in the expression and activity of drug metabolizing enzymes, transporters and their regulators is a major determinant of drug response, both in terms of drug efficacy and adverse events. The cytochrome P450 enzymes, a superfamily of microsomal and mitochondrial hemoproteins (CYP), are particularly variable between and within subjects (Zanger and Schwab, 2013). Despite half a century of pharmacogenetic research, much of the interindividual variation remains unexplained (Meyer, 2004; Lauschke and Ingelman-Sundberg, 2015). This “dark variation” may be explained by so far unidentified or unexplored genes that influence drug metabolism at the level of protein-protein interactions (PPI). The multiple monooxygenase functions of microsomal CYP enzymes strictly depend on PPI with the single microsomal flavoprotein NADPH:cytochrome P450 oxidoreductase (POR) to allow electron transfer from NADPH to the heme iron (Pandey and Fluck, 2013; Kandel and Lampe, 2014). Since POR is present in the endoplasmic reticulum (ER) at sub-stoichiometric amounts compared to CYP, it has been proposed that their interactions involve transient oligomeric complexes (Backes and Kelley, 2003). In contrast to POR, the small hemoprotein cytochrome b5 (CYB5), which is located together with CYB5-reductase and the other components on the cytoplasmic side of the ER, is not an obligatory electron donor for CYPs but acts as modulator of enzyme activity in CYP- and sometimes substrate-dependent way (Henderson et al., 2015). For a long time, POR and CYB5 were the only proteins known to functionally interact with CYPs during the microsomal monooxygenase reaction. Only in recent years new PPI candidates for cytochromes P450 have been considered (see other articles in this Research Topic).

A particularly intriguing group of proteins are the so-called “membrane-associated progesterone receptor” (MAPR) proteins. The term emphasizes their common ability to bind progesterone and to elicit rapid steroidal effects independent of the classical steroid hormone receptors (Wehling, 1997; Mifsud and Bateman, 2002). Following the initial purification of a high-affinity progesterone-binding protein (later termed progesterone receptor membrane component 1, PGRMC1) from porcine liver membranes and cDNA cloning (Meyer et al., 1996; Gerdes et al., 1998), MAPRs were recognized as evolutionarily conserved, distant homologs of CYB5, that can principally interact with CYPs and modulate their functions (Min et al., 2004; Hughes et al., 2007).

The purpose of this review is to provide an overview and critical assessment of the studies elucidating potential interactions of MAPRs with CYPs. Apart from this, MAPRs interact with an increasing number of different proteins and are involved in a wide variety of cellular functions including cholesterol and steroid homeostasis, cell cycle regulation, cell migration, neurogenesis, autophagy, heme homeostasis and more that are only briefly mentioned but not the focus of this review. Readers interested in these aspects are referred to excellent reviews by others (Losel et al., 2008; Ahmed et al., 2012; Kimura et al., 2012; Neubauer et al., 2013; Cahill et al., 2016; Hasegawa et al., 2016).

## Molecular, Biochemical and Cellular Properties of Maprs

The four MAPR proteins PGRMC1, PGRMC2, neuferricin and neudesin share a homologous CYB5-like heme/steroid-binding domain but lack homology with the classical nuclear or membrane-bound steroid receptors (Mifsud and Bateman, 2002; Min et al., 2005; Kimura et al., 2012). Recent crystallographic analyses confirmed the hemoprotein nature of PGRMC1 with a special five-coordinated heme iron involving Tyr113, as opposed to the six-coordinated heme with two axial histidines in CYB5 (Min et al., 2005; Kaluka et al., 2015; Kabe et al., 2016). Interestingly, this difference allows human PGRMC1 to form stable homodimers through hydrophobic heme-heme stacking interactions, explaining earlier observations of dimeric complexes (Min et al., 2004; Kabe et al., 2016). Since the heme-binding residues are conserved among MAPRs, these proteins may all share the ability of heme-dependent homo- and/or heterodimerization (Peluso et al., 2014). Although PGRMC1 had originally been defined as a component of a progesterone-binding protein complex with affinities in the nM range (Meyer et al., 1996), it remained questionable whether progesterone actually binds directly to PGRMC1 itself. A recent study provided first qualitative spectroscopic evidence that this is indeed the case, while it is still not proven for other MAPRs (Kaluka et al., 2015).

### PRGMC1

PRGMC1 is predominantly expressed in mammalian liver and kidney but also found in steroidogenic and reproductive tissues, brain, breast, heart, lung, skeletal muscle, pancreas, and other organs (Gerdes et al., 1998; Kimura et al., 2012). Typically it is colocalized with CYPs in the smooth ER, but it has also been localized in the nucleus, cytoplasm, plasma membrane, and mitochondria (Kimura et al., 2012; Piel et al., 2016). Expression is inducible by carcinogens, including dioxin (Selmin et al., 1996), and increased in breast and other tumors, where it contributes to cancer progression (Neubauer et al., 2008; Ruan et al., 2017). Reported physiological functions affected by PGRMC1 include cholesterol/steroid biosynthesis and metabolism (see below), iron homeostasis and heme trafficking by regulating hepcidin expression and ferrochelatase activity (Craven et al., 2007; Li et al., 2016; Piel et al., 2016), promotion of autophagy through interaction with MAP1LC3B (Mir et al., 2013), regulation of cell cycle, proliferation, and cell death (Peluso et al., 2014), maintenance of female reproductive functions in PGRMC1 conditional knock-out mice (McCallum et al., 2016), cell migration and invasion (Sueldo et al., 2015), amyloid beta binding and synaptotoxicity in a mouse model of Alzheimer’s disease (Izzo et al., 2014), and others (Losel et al., 2008; Ahmed et al., 2012; Neubauer et al., 2013; Cahill et al., 2016; Hasegawa et al., 2016). A growing number of studies supports furthermore a role of PGRMC1 in drug response and as potential drug target, e.g., by increasing susceptibility to tyrosine kinase inhibitors (Ahmed et al., 2010), decreasing doxorubicin cytotoxicity (Lin et al., 2015), or mediating atypical antipsychotic drug-induced lipid disturbances (Cai et al., 2015). Importantly, in 2011 PGRMC1 was identified as the elusive sigma 2 receptor (S2R), an intracellular orphan receptor that binds to many drugs and that is abundant in liver and kidney (Xu et al., 2011; Ahmed et al., 2012).

### PGRMC2

PGRMC2 harbors a structurally similar CYB5 domain as PGRMC1, while there are differences in the N-terminal transmembrane domain (Cahill, 2007). Human PGRMC2 protein is 247 amino acids in length. Despite its structural similarities to PGRMC1, PGRMC2 has been less well studied (Wendler and Wehling, 2013; Hasegawa et al., 2016). Expression appears to be ubiquitous with similar intracellular localization (Chen et al., 2010; Intlekofer and Petersen, 2011). In SKOV-3 ovarian cancer cells no differences between PGRMC1 and 2 in regard to cell viability or response to cisplatin and progesterone were found, but only PGRMC2 inhibited cell migration (Albrecht et al., 2012). PGRMC2 was also implicated in cell cycle regulation (Peluso et al., 2014).

### Neudesin

Neudesin was originally identified as a mouse secreted protein with neurotrophic activity and termed neuron-derived neurotrophic factor (NENF) (Kimura et al., 2005). In adult mice, neudesin was expressed preferentially in the CNS spinal cord but also in several peripheral tissues (Kimura et al., 2005, 2012). NMR studies suggested a similar structure with a potential heme-binding pocket (Han et al., 2012). The physiological roles of neudesin as a secreted protein appear to be significantly different from PGRMC1/2, including particularly neurotrophic activity that required heme binding and was mediated through mitogen-activated protein (MAP) and phosphatidylinositol 3-kinase (PI-3K) pathways (Kimura et al., 2005). Several neurological functions have been studied in knockout mice as summarized elsewhere (Ohta et al., 2015). Extracellular/secreted neudesin may be involved in carcinogen resistance and tumor cell immortalization, although specific receptors remain unknown (Ohta et al., 2015).

### Neuferricin

Neuferricin (CYP5D2) was discovered *via* a homology-based search of the CYB5-like heme/steroid-binding domain of neudesin, alternatively termed cytochrome B5 Domain Containing 2 (CYB5D2) (Kimura et al., 2010). Neuferricin is also widely expressed in several tissues including the CNS, heart, adrenal glands, and kidneys. Human neuferricin is 264 amino acids in length and includes a cleavable signal sequence at its N terminus followed by a heme-binding domain that may involve aspartic acid (D86) instead of tyrosine in heme binding (Bruce and Rybak, 2014). CYB5D2 can be detected as secreted hemoprotein in some cell lines but also colocalized with POR in the ER (Xie et al., 2011; Bruce and Rybak, 2014). Ectopic CYB5D2 expression inhibited cell proliferation and anchorage-independent colony growth of HeLa cells (Bruce and Rybak, 2014).

## Specific Involvement of Maprs in CYP Functions

**FIGURE 1:**
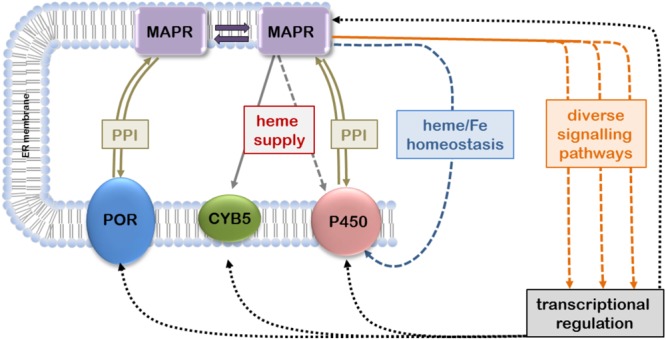
**Confirmed and hypothetical interactions of membrane-associated progesterone receptors (MAPRs) with microsomal cytochrome P450 (CYP) system.** MAPR proteins located in the endoplasmic reticulum (ER) membrane can directly interact with each other to form homo- and heterodimers; MAPR monomers and dimers may participate in PPIs with P450 oxidoreductase (POR), cytochrome b5 (CYB5) and/or CYPs. Transfer of heme to CYB5 and possibly CYPs as well as involvement in iron/heme homeostasis may influence heme integration and thus CYP function. Furthermore, MAPRs participate in diverse signaling pathways that may affect transcriptional regulation of CYP gene expression (double arrows, confirmed protein-protein interactions; solid arrows, confirmed influence; dashed arrows, hypothetical influence).

The available evidence suggesting that MAPR proteins can interact with the microsomal CYP-monooxygenase system to modulate its function is based on different species from yeast to human, various experimental systems, and relates to CYP enzymes catalyzing steroidogenic or xenobiotic reactions (**Table 1**). The levels of interactions may include not only physical PPIs but also indirect influences via heme transfer, heme and iron homeostasis, and transcriptional regulation (**Figure 1**).

**Table 1:** Functional interactions between membrane-associated progesterone receptors (MAPR) proteins and cytochrome P450 system.

MAPR	CYP	System	Enzymatic activity	Direction of influence	Reference
IZA/PGRMC1 (rat)	CYP21A2 (rat)	Inhibition by anti-IZA monoclonal antibody	Progesterone 21-hydroxylase	Activation	Laird et al., 1988
IZA/PGRMC1 (rat)	CYP21A2 (rat)	COS-7 cell coexpression	Progesterone 21-hydroxylase	Activation	Min et al., 2004
IZA/PGRMC1 (rat)	CYP17 (guinea pig)	COS-7 cell coexpression	Progesterone 17α-hydroxylase	Little or no influence	Min et al., 2005
IZA/PGRMC1 (rat)	CYP17 (guinea pig)	COS-7 cell coexpression	17α-Hydroxyprogesterone 17–20 lyase	Activation	Min et al., 2005
IZA/PGRMC1 (rat)	CYP11B1 (rat)	COS-7 cell coexpression	Progesterone 11β-hydroxylase	Little or no influence	Min et al., 2004
PGRMC1 (human)	CYP19A1 (human)	CYP19-engineered MCF-7 human breast cancer cells RNAi knockdown	Androst-4-ene-3,17-dione conversion	Activation	Ahmed et al., 2012
Dap1/PGRMC1 (*S. cerevisiae*)	CYP51A1 (*S. cerevisiae*)	*S. cerevisiae* genetics	Lanosterol-14-demethylase	Activation	Hand et al., 2003 Mallory et al., 2005
Dap1/PGRMC1 (*S. pombe*)	CYP51A1 (*S. pombe*)	*S. pombe* strain lacking DAP1	Lanosterol-14-demethylase	Activation	Hughes et al., 2007
Dap1/PGRMC1 (*S. pombe*)	CYP61A1 (*S. pombe*)	*S. pombe* strain lacking DAP1	Lanosterol-22-desaturase	Activation	Hughes et al., 2007
PGRMC1 (rabbit)	CYP2C2 (rabbit)	HEK293, HepG2 cell coexpression	Luciferin 6′ methyl ether *O*-demethylation	Inhibition	Szczesna-Skorupa and Kemper, 2011
PGRMC1 (human)	CYP2C8 (human)	HEK293, HepG2 cell coexpression	Luciferin 6′ methyl ether *O*-demethylation	Inhibition	Szczesna-Skorupa and Kemper, 2011
PGRMC1 (human)	CYP3A4 (human)	HEK293, HepG2 cell coexpression	Luciferin 6′ pentafluorobenzyl ether depentafluorobenzylation	Inhibition	Szczesna-Skorupa and Kemper, 2011
PGRMC1 (human)	CYP2C9 (human)	HepG2 coexpression, human hepatocytes RNAi knockdown	*S*-Warfarin 7-hydroxylase and diclofenac 4′-hydroxylase	Inhibition	Oda et al., 2011
PGRMC1 (human)	CYP3A4 (human)	HepG2 coexpression, human hepatocytes RNAi knockdown	Testosterone 6β-hydroxylase and midazolam 1′-hydroxylase	Inhibition	Oda et al., 2011
PGRMC1 (human)	CYP2E1 (human)	HepG2 coexpression, human hepatocytes RNAi knockdown	Chlorzoxazone 6-hydroxylase and 7-ethoxycoumarin *O*-deethylase	No influence	Oda et al., 2011
PGRMC2 (human)	CYP3A4 (human)	Human liver genetic association	Atorvastatin 2-hydroxylase	Inhibition	Klein et al., 2012
CYB5D2 (human)	CYP3A4 (human)	Hela cell expression and RNAi knockdown	Luciferin 6′ pentafluorobenzyl ether depentafluorobenzylation	Activation	Bruce and Rybak, 2014

### PGRMC1

First indications for a link between PGRMC1 and CYP function came from two independent studies. Identification of the adrenal “inner zone antigen” (IZA) as a MAPR member (Raza et al., 2001), its localization in both steroidogenic and steroid metabolizing tissues and the fact that an anti-IZA monoclonal antibody decreased certain adrenal steroidhydroxylase activities led the authors to suggest a function in steroid hormone biosynthesis and/or metabolism (Min et al., 2004). By coexpressing IZA/PGRMC1 with CYP21A2, CYP11B1 or CYP17 in COS-7 cells they found differential effects, namely enhancement of progesterone 21-hydroxylation, no effect on progesterone 11β- or 17α-hydroxylation, and activation of the CYP17-catalyzed 17–20 lyase reaction (Min et al., 2004, 2005). About at the same time a search for novel yeast genes involved in DNA damage response led to the identification of *Dap1* (Hand et al., 2003). Steroid analyses of *Saccharomyces cerevisiae* yeast cells lacking *Dap1* revealed decreased levels of ergosterol but increased levels of its intermediates, suggesting a partial defect in sterol synthesis by lanosterol-14-demethylase, the highly conserved yeast-essential Erg11/CYP51 (Hand et al., 2003). Genetic follow-up studies supported a model in which heme binding by Dap1 is required to activate Erg11/CYP51, although a Dap1-Erg11 complex could not be directly detected by immunoprecipitation experiments (Mallory et al., 2005).

Further evidence for a role of Dap1/PGRMC1 in CYP function was provided by Hughes et al. (2007) working with *Schizosaccharomyces pombe*. After confirming that fission yeast Dap1 is also a hemoprotein and directly binds to and activates enzymatic activities of CYP51A1 (Erg11) and CYP61A1 (Erg5), they investigated whether PGRMC1 is required for cholesterol synthesis in mammals (Hughes et al., 2007). RNA interference-mediated knock-down of hPGRMC1 in HEK293 cells resulted in lanosterol accumulation, indicating that PGRMC1 was required for the demethylation of lanosterol by the Erg11 homolog, CYP51A1. Using a coexpression/coimmunoprecipitation approach PGRMC1 was shown to form a stable complex with CYP51A1 (Hughes et al., 2007). The authors applied the latter experiment also to CYP7A1, CYP21A2, and CYP3A4, demonstrating physically stable PGRMC1-CYP complexes in all cases. PGRMC1 was later shown to also activate CYP19 aromatase (Ahmed et al., 2012). Collectively these studies strongly suggest the ability of PGRMC1 to form functional PPIs with steroidogenic and possibly other CYPs. Interestingly, the documented functional influence of PGRMC1 on various steroidogenic CYPs appeared to be generally positive.

The above mentioned ability of PGRMC1 to complex with CYP3A4 first indicated the possibility that PGRMC1 may also interact with drug metabolizing P450s (Hughes et al., 2007). Although the authors emphasized the apparent existence of stoichiometric and physically stable complexes, it should be kept in mind that the experimental conditions of overexpressing tagged and manipulated proteins were quite artificial and that it is unknown, whether PGRMC1 abundance in the ER is sufficient for the formation of 1:1 complexes at higher concentrations.

Two studies have investigated the question whether PGRMC1 affects drug metabolizing CYPs functionally. Szczesna-Skorupa and Kemper, using cotransfection and coimmunoprecipitation of FLAG-tagged PGRMC1 with variously tagged rabbit CYP2C2 and human CYP2C8 and CYP3A4 in HEK293 cells could show efficient binding of PGRMC1 to all three CYPs, for CYP2C2 mainly mediated by the cytoplasmic domain (Szczesna-Skorupa and Kemper, 2011). The effect of PGRMC1 on enzymatic activity was tested by siRNA-mediated downregulation of endogenous PGRMC1, and by coexpression with functional CYP plasmids in HEK293 and HepG2 hepatoma cells (**Table 1**). Surprisingly, the results were quite opposite to those of steroidogenic CYPs, as CYP activities were either unchanged or slightly increased in PGRMC1-deficient cells, but decreased in presence of exogenously expressed PGRMC1. Interestingly, the inhibitory effect could be partially and CYP isoform-dependently reversed by increased expression of POR, also shown to directly interact with PGRMC1. The authors concluded that in contrast to steroidogenic CYPs, PGRMC1 is not required by drug metabolizing CYPs for enzymatic activity and that the CYP differential effects may be explained by the intricate PPI affinities in triple CYP-POR-PGRMC1 systems (Szczesna-Skorupa and Kemper, 2011).

Oda et al. (2011) found differential effects of PGRMC1 cotransfection with CYP2C9, CYP2E1, and CYP3A4 in HepG2 cells. While CYP3A4 had increased *K*_m_ and decreased *V*_max_ with two different substrates (testosterone and midazolam), CYP2C9 *K*_m_ was unchanged but *V*_max_ also decreased, and CYP2E1 activity (chlorzoxazone, 7-ethoxycoumarin) remained unchanged. The authors also confirmed direct interactions of PGRMC1 with all three CYPs (Oda et al., 2011).

More recently, Kabe et al. (2016) in their structural study investigated the relevance of heme-mediated dimerization on functional interactions with human CYPs. By incubation of FLAG-tagged PGRMC1 with microsomes from insect-cells expressing POR, CYB5, and either CYP1A2 or CYP3A4, they showed by immunoprecipitation that only wild-type PGRMC1 but not the Y113F mutant interacted with the present CYP. The interaction with CYP1A2 was furthermore blocked by incubation with a CO-generating reagent, indicating that PGRMC1 dimerization is necessary for the interaction. To test whether CYP-mediated enzymatic functions are affected they used HCT116 human colon cancer cells stably expressing control or PGRMC1 shRNA to reduce endogenous PGRMC1 expression and incubated them with doxorubicin. PGRMC1 knock-down suppressed the conversion of doxorubicin to doxorubicinol, and increased cell sensitivity to doxorubicinol. This effect was partially reversed by coexpression of the wild-type PGRMC1 but not of the Y113F mutant. Assuming that CYP2D6 and CYP3A4 are the relevant enzymes for doxorubicinol-formation in HCT116 cells, the authors concluded that interaction of CYPs with the PGRMC1 dimer was a crucial component of CYP activity (Kabe et al., 2016). However, this conclusion is severely flawed because the metabolic step from doxorubicin to doxorubicinol is not a hydroxylation but in fact a 2-electron reduction of a ketogroup to an aliphatic alcohol. This reduction is not catalyzed by CYPs 2D6 and 3A4 as indicated in the paper, but mainly by cytosolic carbonyl reductase 1 (Kassner et al., 2008). Furthermore the use of HCT116 cells with very low CYP3A4 expression seems inappropriate for CYP metabolism studies (Habano et al., 2011). We therefore believe that this and other previously reported functional and cellular effects of PGRMC1 on doxorubicin-metabolism or cytotoxicity (e.g., Friel et al., 2015; Lin et al., 2015) are not due to interactions with CYPs but must have other reasons.

Finally the recent study by Piel et al. (2016) should be mentioned here again as it demonstrated a role of PGRMC1 in heme homeostasis and its ability to directly transfer heme to CYB5 *in vitro*. The authors suggest that PGRMC1 may be a heme chaperone or sensor, and this could of course have additional direct or indirect implications for CYP function (**Figure 1**).

### PGRMC2

Despite its high similarity to PGRMC1 much less is known about PGRMC2 (Wendler and Wehling, 2013). The first observation indicating a role in CYP function was made in a pharmacogenetic screen in human livers, where an intronic PGRMC2 polymorphism (rs3733260) was significantly associated with lower levels of mRNA expression, protein and enzyme activity (Klein et al., 2012). Although the directionality of this influence was the same as that observed for PGRMC1 and CYP3A4, it should be noted that the data are correlative in nature and would also be compatible with gene regulatory mechanisms (**Figure 1**). Stable physical interactions of PGRMC2 were demonstrated in human embryonic kidney cells for CYP1A2 and CYP3A4, suggesting similar interaction potential as for PGRMC1 (Albrecht et al., 2012). It should thus be interesting to investigate the potential of PGRMC2 to modulate CYP function and/or expression in a valid system.

### Neudesin and Neuferricin

To our knowledge, only one study (Bruce and Rybak, 2014) directly addressed the potential interaction of these proteins with CYPs. The authors showed that neuferricin is colocalized with POR in HeLa cells. Neuferricin knockdown reduced endogenous CYP51A1 (lanosterol demethylase) protein levels, which lead to increased sensitivity toward mevalonate, an intermediate of the cholesterol synthesis pathway. This suggested a stabilizing role of neuferricin for CYP51A1, as previously described for PGRMC1 and Dap1 (Hughes et al., 2007). Data further indicated that neuferricin knockdown also decreased the fraction of endogenous CYP3A4 activity that was enhanced by overexpressed POR, indicating activation of CYP3A4 by neuferricin, in contrast to the inhibitory effects of PGRMC1 (see above). However, it should be pointed out that the neuferricin data consists of a single point measurement carried out in a cell model that is not usually applied for drug metabolism studies, leaving it unclear whether the conclusion can be generalized. The same argument applies to the studies on HeLa cell exposure to chemotherapeutic agents such as paclitaxel, cisplatin, and doxorubicin, which resulted in increased sensitivities in CYB5D2 deficient cells (Bruce and Rybak, 2014). Since the expression levels of drug metabolizing CYPs are very low in HeLa cells, the construction of a link between CYB5D2 and function of drug metabolizing CYPs based on these data is in our opinion questionable.

## Conclusion and Future Perspectives

Direct physical interactions of MAPRs, especially of mammalian PGRMC1 or yeast Dap1, with various cytochromes P450 are strongly supported by experimental data. However, data are still limited and in our view use of inappropriate model systems questions some conclusions on functional interactions with CYPs. Open questions that remain to be studied are, for example: which direct MAPR-CYP interactions are possible in binary, ternary, and higher order complexes and how do they affect function; how could MAPRs indirectly influence CYP function, e.g., by regulating heme supply and protein stability; are there MAPR signaling pathways that affect transcriptional regulation of CYPs and possibly other drug metabolizing enzymes, etc. It is of eminent importance that valid experimental systems, which express the CYP system at physiological levels, e.g., primary hepatocytes or genetically modified mice, are used in future studies.

## Author Contributions

All authors listed have made substantial, direct and intellectual contribution to the work, and approved it for publication.

## Conflict of Interest Statement

The authors declare that the research was conducted in the absence of any commercial or financial relationships that could be construed as a potential conflict of interest.
